# Supplementary material for: SecA2 Associates with Translating Ribosomes and Contributes to the Secretion of Potent IFN-β Inducing RNAs
Source: Int J Mol Sci. 2022 Nov 30;23(23):15021. doi: 10.3390/ijms232315021 (PMC9736482; doi:10.3390/ijms232315021)
Supplement: Supplementary file 1 [file ijms-23-15021-s001.zip › ijms-1924373-supplementary.pdf]

Supplementary Material

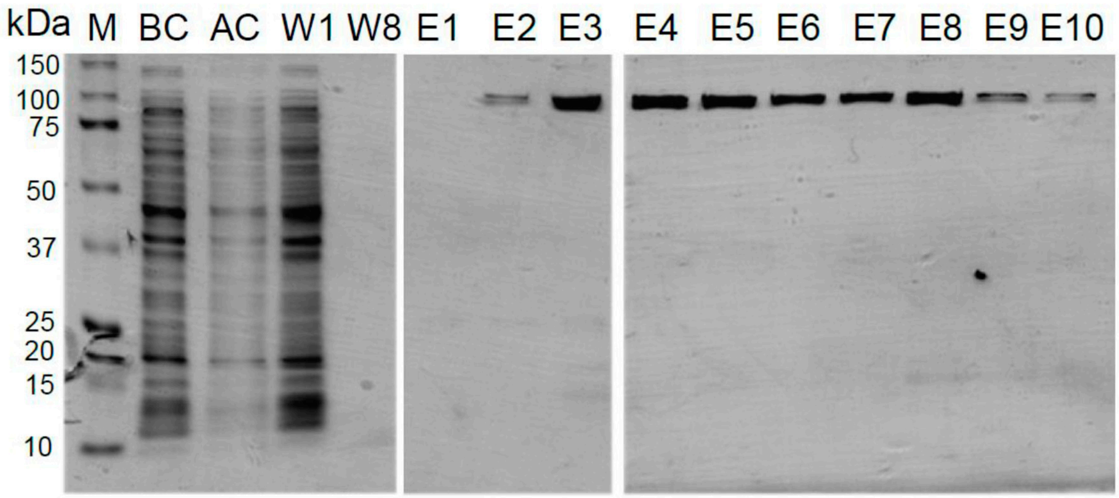

Figure S1. Affinity chromatography for SecA2. Protein patterns of cytosolic fractions before (before column, BC) and after (after column, AC) pass through the streptactin affinity chromatography column. Coomassie gel of wash fractions (the first W1 and the eighth W8) and the elution fractions (E1 to E10) are depicted.

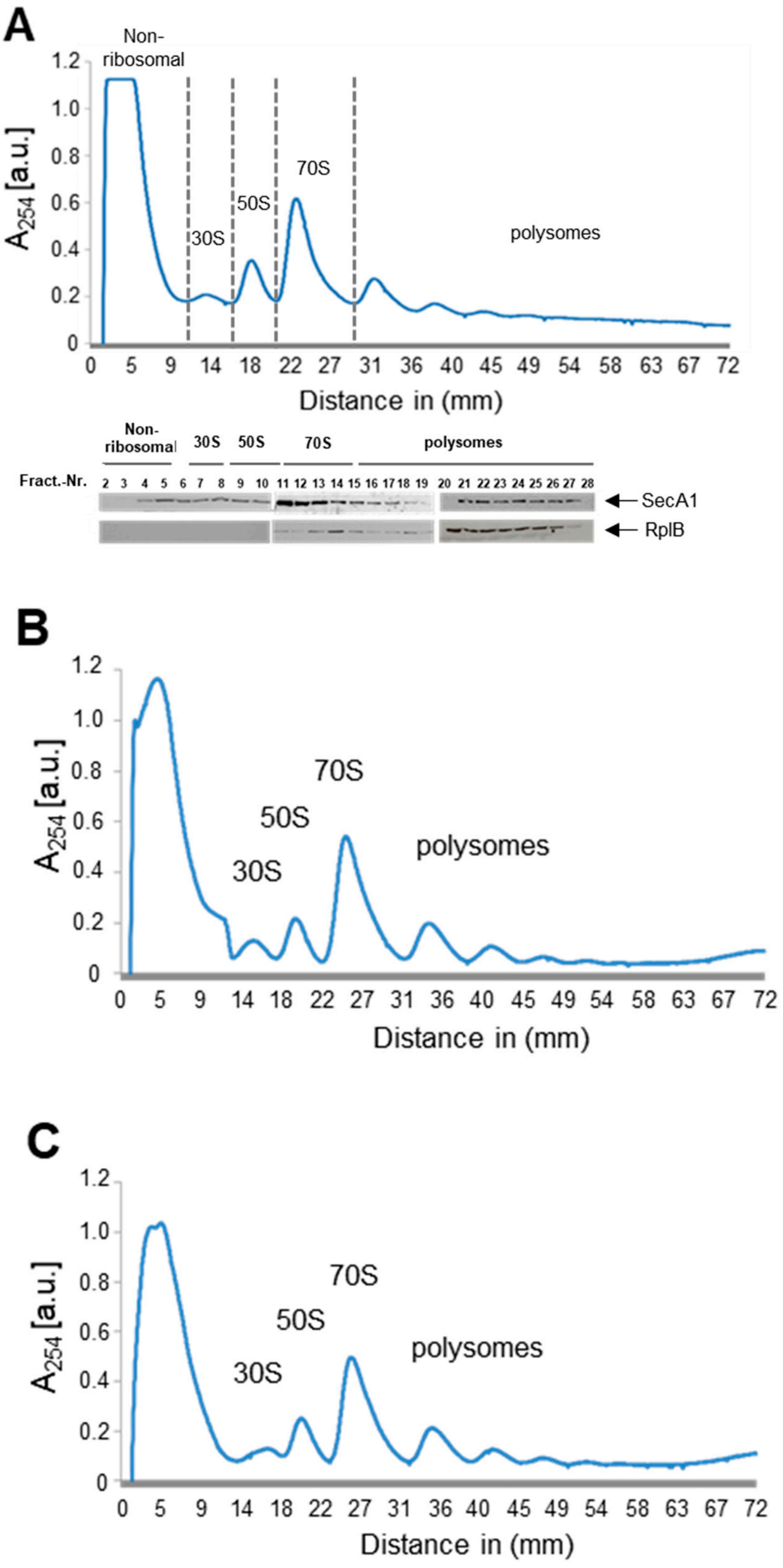

Figure S2. Ribosome profiles of recombinant SecA1 expressing strain Lm-SecA1 (A), wild type strain (B) and *L. monocytogenes* harboring the empty pERL-3 vector (C). Ribosomal peaks were normalized to the same gradient baseline by subtracting the area under the polysome profile for quantitative comparison.

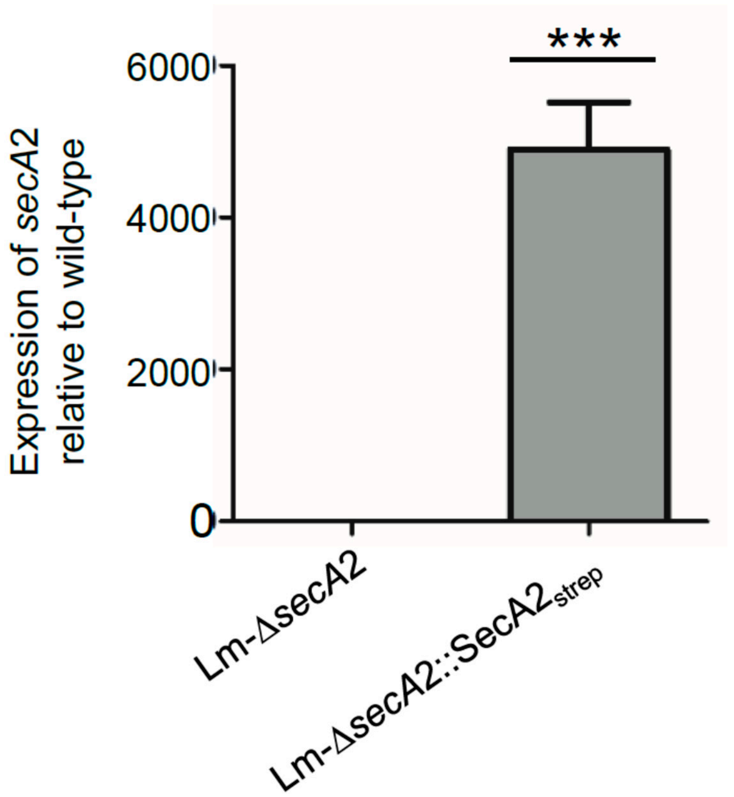

Figure S3. Expression of *secA2* in Lm-Δ*secA2* and the complemented strain Lm-Δ*secA2*::SecA2$_{strep}$ as compared to wild-type. The transcript amounts were determined by using qRT-PCR. Specific secA2 primers are listed in table S1.

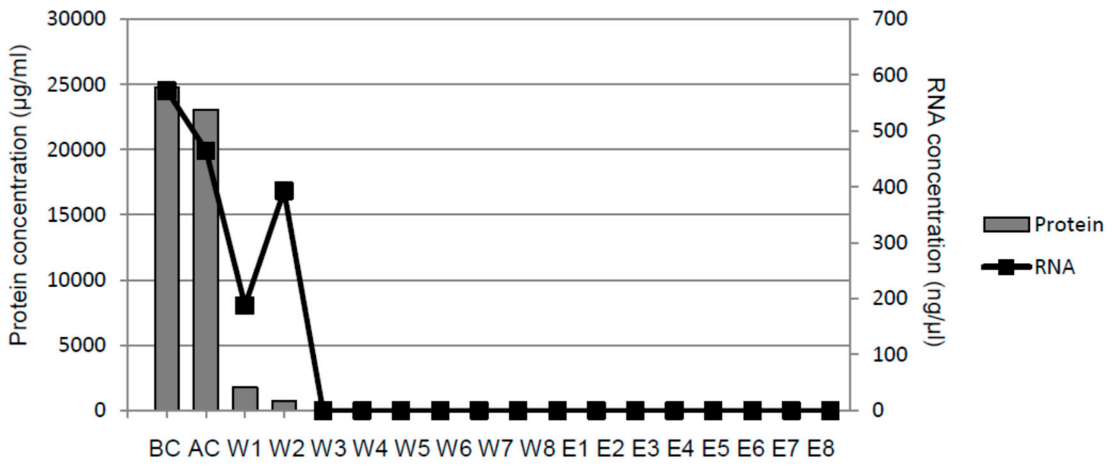

Figure S4. Affinity chromatography of lysates from *L. monocytogenes* wild type strain without affinity-tagged protein expressing pERL-3 plasmid. Neither protein nor RNA were elutable.

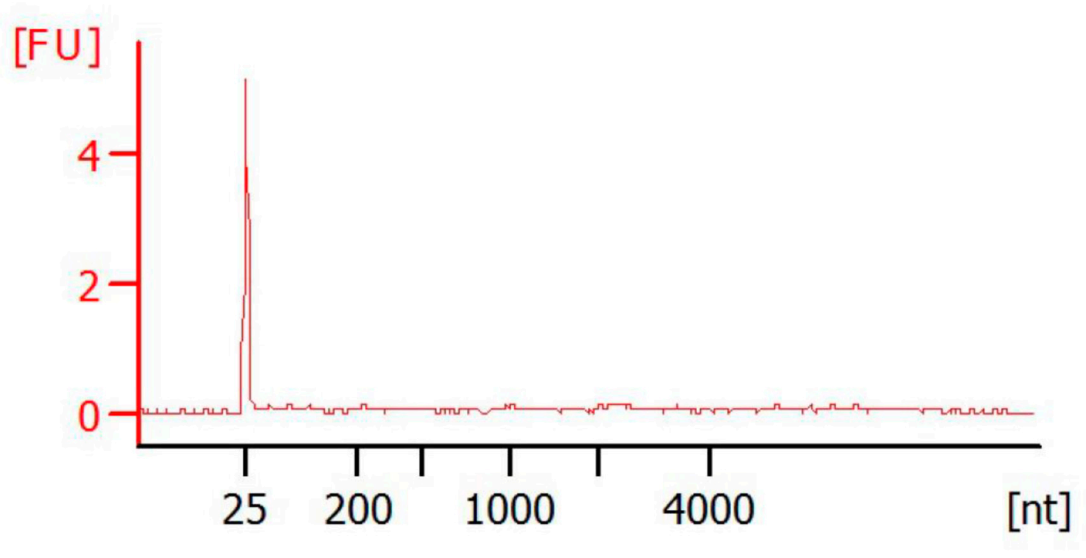

Figure S5. RNase I treatment SecA2- and SecA1-associated RNAs are completely digested.

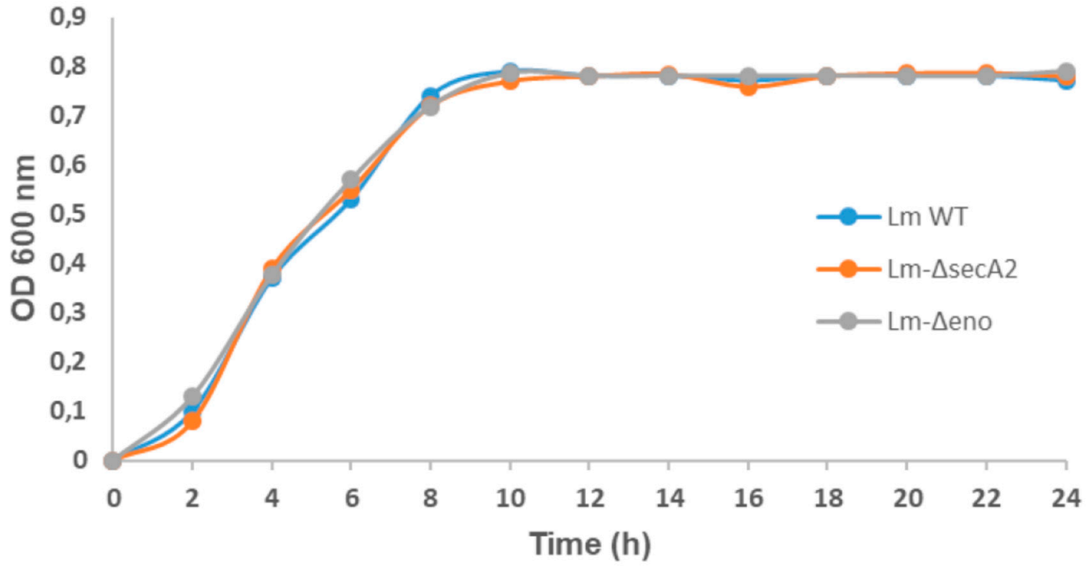

Figure S6. In-vitro growth of *L. monocytogenes* wild type (Lm WT), Lm-Δ*secA2* and Lm-Δ*eno* at 37°C in BHI. Values represent one of three independent experiments.

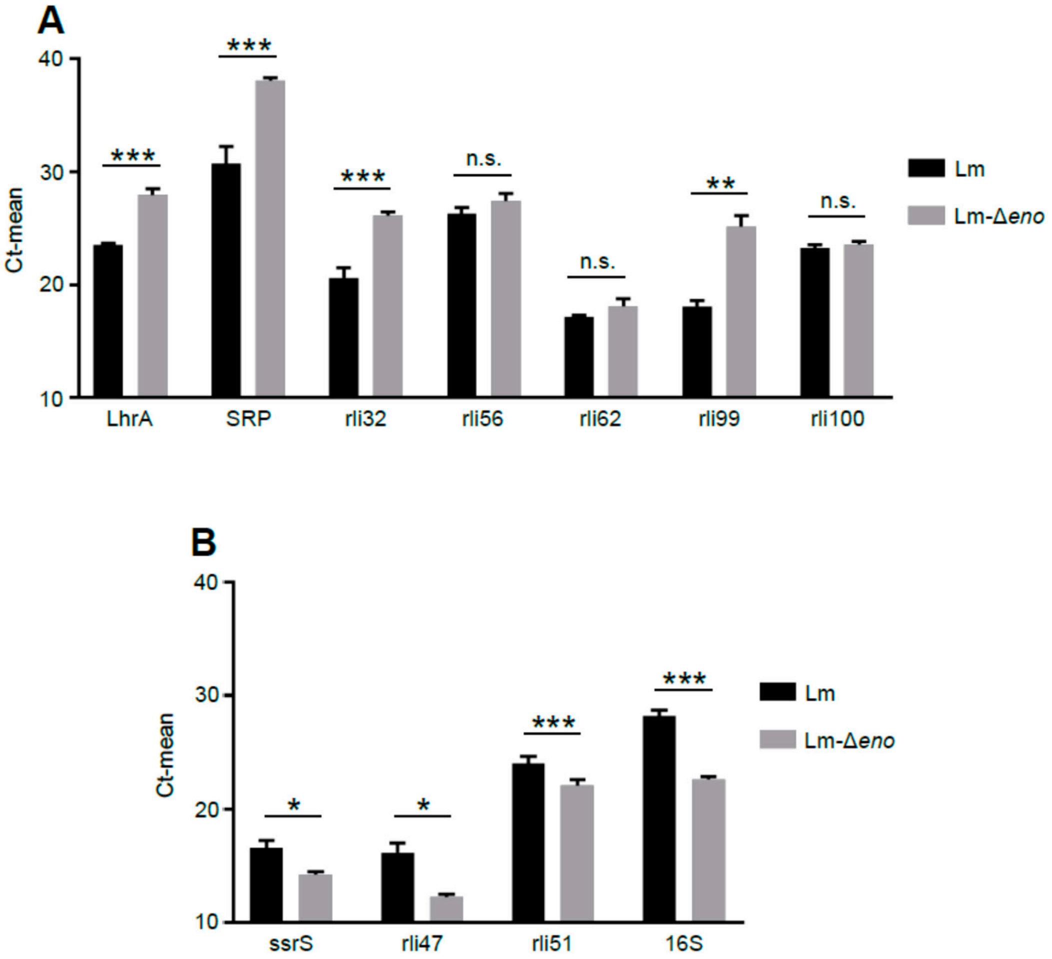

Figure S7. (A) Impact of enolase deletion on the transcript amounts of selected sRNAs in sec-RNA of Lm and Lm-Δ*eno*. The amounts of the sRNAs rli32, LhrA, SRP and rli99 are decreased in Lm-Δ*eno*, whereas the amounts of rli56, rli62, rli100 are unchanged. The transcript amounts in sec-RNA were determined by using qRT-PCR. The data represent the mean of three independent experiments (n.s. not significant). (B) sRNAs (ssrS, ri47, rli51 and 16S rRNA) whose amounts were found to be increased in the absence of enolase. Data are presented as means ± SD of results from three experiments (ns, nonsignificant; * P < 0.05, ** P < 0.01; *** P < 0.001).

Table S1. List of SecA2-dependent proteins according to Lenz et al. (2003) and Renier et al. (2014). Identified proteins that were co-isolated with SecA2 in this study are highlighted in blue.

Lmo Nr.	Gene name	Protein name
lmo0258	rpoB	RNA polymerase beta subunit
lmo0259	rpoC	DNA-directed RNA polymerase subunit beta'
lmo0582	p60	Invasion associated protein (Iap), Cell-wall hydrolase
lmo1054	pdhC	Dihydrolipoamide acyltransferase (E2)
lmo1388	tcsA	CD4+ T-cell stimulating antigen
lmo1439	sodA	Superoxide dismutase
lmo1473	dnaK	Chaperone protein DnaK
lmo1634	lap	Bifunctional aldehyde/alcohol dehydrogenase; listerial adhesion protein
lmo1829	fbpA	Fibronectin-binding protein A
lmo2039	FtsI	Cell division protein FtsI
lmo2068	groEL	60 kDa chaperonin
lmo2110		Mannose-6-phosphate isomerase
lmo2125		ABC-type maltose transporter
lmo2196	oppA	ABC-type oligopeptide transport system
lmo2455	eno	Enolase
lmo2526	murA	UDP-N-acetylglucosamine enolpyruvyl transferase
lmo2596	rpsI	30S ribosomal protein S9
lmo2637	pplA	Peptide pheromone-encoding lipoprotein A, NADH-(ubi-)quinone oxireductase subunit
lmo2653	tuf	Elongation factor Tu

Table S2. Primers used in this study (5'- to 3'-prime direction).

Phelp-for.	gcgcgatctcgagatctgcaggat
LT_StrepLink_rev	atagtccagctgctgctttttcaaattgtgg
LT_SecStrep_for	aaaagcagcagctggactattga
LT_SecA_Term_rev	acttttacaactatgcttctttac
LT_SecA_Term_for	agaagcatagttgtaaaagtaataaaaaattaag
Term.-rev.	gcgccccggggcttatattatat
lmo2455_1r	aaaaaggtgtaacgcttcgtg
lmo2455_2f	aatcaactta_ttataattctctcctttgtt
lmo2455_3r	agaattataa_taagttgattgaataaaacg
lmo2455_4f	gcgcaccgtttatttcataag
secA2 f	cggcgctttaatgatggactccatc
secA2 r	gacggaattcttcttcctcag

Table S3. SecA2 associated sRNAs

Name	Chromosome	Region	Expression value	Gene length	RPKM	Unique gene reads	Total gene reads
ssrA	EGDe (NCBI)	complement(2509737..2510236)	29485,6787	500	29485,6787	21876	21876
SRP	EGDe (NCBI)	complement(2784206..2784540)	7930,21627	335	7930,21627	3942	3942
ssrS	EGDe (NCBI)	complement(1546330..1546537)	1892,18123	208	1892,18123	584	584
rli61	EGDe (NCBI)	complement(2275258..2275362)	2631,52667	105	2631,52667	410	410
rli47	EGDe (NCBI)	2226036..2226349	862,798981	314	862,798981	402	402
rli32	EGDe (NCBI)	complement(600602..600751)	1010,89134	150	1010,89134	225	225
rli33-1	EGDe (NCBI)	708309..708494	474,647907	186	474,647907	131	131
rli31	EGDe (NCBI)	597806..597949	421,204726	144	421,204726	90	90
rli90	EGDe (NCBI)	complement(1527932..1528048)	489,605494	117	489,605494	85	85
rnpB	EGDe (NCBI)	complement(1961656..1961974)	147,883791	319	147,883791	70	70
rli48	EGDe (NCBI)	complement(2361297..2361404)	380,644271	108	380,644271	61	61
rli72	EGDe (NCBI)	200410..200519	238,937954	110	238,937954	39	39
rli56	EGDe (NCBI)	1199848..1199937	292,035277	90	292,035277	39	39
LhrA	EGDe (NCBI)	complement(2346167..2346434)	95,5568932	268	95,5568932	38	38
rli50	EGDe (NCBI)	complement(2782959..2783264)	70,4760849	306	70,4760849	31	32
sbrA	EGDe (NCBI)	1399365..1399464	175,221166	100	175,221166	26	26
rli88	EGDe (NCBI)	1320429..1320509	183,042054	81	183,042054	22	22
rli97	EGDe (NCBI)	complement(2280060..2280111)	272,163054	52	272,163054	21	21
rli114	EGDe (NCBI)	complement(2857244..2857320)	175,04612	77	175,04612	20	20
rli111	EGDe (NCBI)	2736465..2736564	121,306961	100	121,306961	18	18
rli86	EGDe (NCBI)	1272592..1272662	142,379062	71	142,379062	15	15
rli28	EGDe (NCBI)	complement(507068..507402)	32,1875851	335	32,1875851	14	16
rli112	EGDe (NCBI)	2782958..2783091	70,4103423	134	70,4103423	14	14
rli91	EGDe (NCBI)	complement(1575830..1575917)	91,899213	88	91,899213	12	12
rli98	EGDe (NCBI)	2361255..2361407	52,8570637	153	52,8570637	12	12
LhrC-1	EGDe (NCBI)	231866..232004	92,1195948	139	92,1195948	11	19
rli77	EGDe (NCBI)	454095..454227	55,7383698	133	55,7383698	11	11
rli62	EGDe (NCBI)	complement(2364285..2364497)	34,8037708	213	34,8037708	11	11
rli78	EGDe (NCBI)	507066..507199	45,2637915	134	45,2637915	9	9
rli53	EGDe (NCBI)	955824..956029	26,1719442	206	26,1719442	8	8
LhrC-5	EGDe (NCBI)	981620..981732	47,7116858	113	47,7116858	8	8
rli92	EGDe (NCBI)	1638942..1639083	33,2217812	142	33,2217812	7	7
rli29	EGDe (NCBI)	complement(507418..507632)	18,8072808	215	18,8072808	6	6
rli81	EGDe (NCBI)	905683..905860	22,7166594	178	22,7166594	6	6
rli87	EGDe (NCBI)	1301269..1301389	33,4178956	121	33,4178956	6	6

rli108	EGDe (NCBI)	complement(2639710..2639789)	42,1204726	80	42,1204726	5	5
rli55	EGDe (NCBI)	1198094..1198610	5,21413975	517	5,21413975	4	4
rli42	EGDe (NCBI)	complement(1399445..1399616)	15,672734	172	15,672734	4	4
rli45	EGDe (NCBI)	2154757..2154883	21,226065	127	21,226065	4	4
rli100	EGDe (NCBI)	complement(2424801..2424875)	35,9428033	75	35,9428033	4	4
rliI	EGDe (NCBI)	complement(2841963..2842200)	11,3265137	238	11,3265137	4	4
LhrC-3	EGDe (NCBI)	232288..232401	17,7349358	114	17,7349358	3	3
rli65	EGDe (NCBI)	531146..531281	14,8660492	136	14,8660492	3	3
rli84	EGDe (NCBI)	1093855..1093954	20,2178269	100	20,2178269	3	3
rli58	EGDe (NCBI)	complement(1639909..1640182)	7,37876893	274	7,37876893	3	3
rli59	EGDe (NCBI)	complement(1702295..1702543)	8,11960918	249	8,11960918	3	3
LhrB	EGDe (NCBI)	complement(1859877..1860018)	14,2379062	142	14,2379062	3	3
rli63	EGDe (NCBI)	complement(2613039..2613300)	7,71672781	262	7,71672781	3	3
rli51	EGDe (NCBI)	207589..207714	10,6972629	126	10,6972629	2	2
LhrC-2	EGDe (NCBI)	232053..232207	8,69583951	155	8,69583951	2	2
LhrC-4	EGDe (NCBI)	232459..232604	9,23188441	146	9,23188441	2	2
rli44	EGDe (NCBI)	2039087..2039362	4,88353306	276	4,88353306	2	2
rli46	EGDe (NCBI)	complement(2154764..2154971)	6,48007271	208	6,48007271	2	2
rli101	EGDe (NCBI)	complement(2452913..2452994)	16,4372576	82	16,4372576	2	2
rli24	EGDe (NCBI)	271014..271201	3,58472108	188	3,58472108	1	1
rli76	EGDe (NCBI)	complement(344675..344772)	6,87681186	98	6,87681186	1	1
rli33-2	EGDe (NCBI)	708618..708891	2,45958964	274	2,45958964	1	1
rli80	EGDe (NCBI)	787038..787317	2,40688415	280	2,40688415	1	1
rli41	EGDe (NCBI)	complement(1276706..1276847)	4,74596875	142	4,74596875	1	1
rli60	EGDe (NCBI)	2054110..2054356	2,72845167	247	2,72845167	1	1
rli106	EGDe (NCBI)	complement(2594654..2594785)	5,10551184	132	5,10551184	1	1
rli109	EGDe (NCBI)	complement(2694473..2694623)	4,46309644	151	4,46309644	1	1
rli71	EGDe (NCBI)	28964..29061	0	98	0	0	0
rli73	EGDe (NCBI)	200939..201035	0	97	0	0	0
rli74	EGDe (NCBI)	209321..209453	0	133	0	0	0
rli75	EGDe (NCBI)	321290..321363	0	74	0	0	0
rli27	EGDe (NCBI)	434817..434947	0	131	0	0	0
rli52	EGDe (NCBI)	complement(552313..552418)	0	106	0	0	0
rli79	EGDe (NCBI)	696818..696880	0	63	0	0	0
rli82	EGDe (NCBI)	complement(910875..910944)	0	70	0	0	0
rli83	EGDe (NCBI)	926922..927039	0	118	0	0	0
rli38	EGDe (NCBI)	1152805..1152917	0	113	0	0	0
rliC	EGDe (NCBI)	1154414..1154674	0	261	0	0	0
rli85	EGDe (NCBI)	complement(1154617..1154762)	0	146	0	0	0
rli89	EGDe (NCBI)	1399490..1399656	0	167	0	0	0
rli93	EGDe (NCBI)	1641179..1641239	0	61	0	0	0
rli94	EGDe (NCBI)	complement(2039254..2039395)	0	142	0	0	0
rli95	EGDe (NCBI)	2106222..2106324	0	103	0	0	0
rli96	EGDe (NCBI)	complement(2239222..2239295)	0	74	0	0	0
rliG	EGDe (NCBI)	complement(2386714..2386829)	0	116	0	0	0
rli99	EGDe (NCBI)	2394985..2395110	0	126	0	0	0
rli102	EGDe (NCBI)	2454489..2454586	0	98	0	0	0
rli103	EGDe (NCBI)	complement(2498270..2498344)	0	75	0	0	0
rli104	EGDe (NCBI)	complement(2505181..2505340)	0	160	0	0	0
rli105	EGDe (NCBI)	complement(2533473..2533615)	0	143	0	0	0
rli107	EGDe (NCBI)	complement(2632700..2632770)	0	71	0	0	0
rli110	EGDe (NCBI)	2730489..2730555	0	67	0	0	0
rli113	EGDe (NCBI)	complement(2839445..2839574)	0	130	0	0	0

Table S4. SecA2 associated mRNAs

Name+A2:H72	Chromosome	Region	Expression value	Gene length	RPKM	Unique gene reads*	Total gene reads*
lmo0583	EGDe (NCBI)	620805..623135	600,4003898	2331	600,40039	2071	2071
lmo0048	EGDe (NCBI)	51775..52389	350,5247655	615	350,524766	319	319
gap	EGDe (NCBI)	complement(2531310..2532320)	157,0796218	1011	157,079622	235	235
lmo0355	EGDe (NCBI)	382016..383536	87,52661891	1521	87,5266189	197	197
lmo2158	EGDe (NCBI)	complement(2242097..2242282)	693,9426228	186	693,942623	191	191
tuf	EGDe (NCBI)	complement(2726008..2727195)	105,2345693	1188	105,234569	185	185
lmo1634	EGDe (NCBI)	1677409..1680009	44,16840428	2601	44,1684043	170	170
lmo2511	EGDe (NCBI)	complement(2590044..2590607)	180,9260007	564	180,926001	151	151
lmo1601	EGDe (NCBI)	complement(1645412..1645936)	193,0790244	525	193,079024	150	150
fbaA	EGDe (NCBI)	complement(2632907..2633761)	117,7669137	855	117,766914	149	149
lmo0197	EGDe (NCBI)	200522..200830	323,6729277	309	323,672928	148	148
glpD	EGDe (NCBI)	1320608..1322284	50,37094406	1677	50,3709441	125	125
lmo1003	EGDe (NCBI)	1033190..1034908	45,99526499	1719	45,995265	117	117
fus	EGDe (NCBI)	complement(2727304..2729391)	37,86679143	2088	37,8667914	117	117
lmo1399	EGDe (NCBI)	1426766..1428328	49,7212459	1563	49,7212459	115	115
rpoD	EGDe (NCBI)	complement(1486480..1487604)	67,27731341	1125	67,2773134	112	112
ctc	EGDe (NCBI)	215721..216344	116,9613321	624	116,961332	108	108
lmo1847	EGDe (NCBI)	complement(1923825..1924757)	75,32772229	933	75,3277223	104	104
fri	EGDe (NCBI)	979059..979529	142,0422123	471	142,042212	99	99
ldh	EGDe (NCBI)	complement(214486..215427)	68,86895139	942	68,8689514	96	96
lmo0913	EGDe (NCBI)	948754..950220	44,22259865	1467	44,2225986	96	96
lmo0539	EGDe (NCBI)	complement(577632..578648)	62,46115933	1017	62,4611593	94	94
lmo1254	EGDe (NCBI)	complement(1278056..1279702)	38,56891259	1647	38,5689126	94	94
lmo1257	EGDe (NCBI)	complement(1281820..1282284)	130,7954682	465	130,795468	90	90
lmo2219	EGDe (NCBI)	2306833..2307714	68,19060784	882	68,1906078	89	89
hup	EGDe (NCBI)	complement(2008319..2008594)	215,4649983	276	215,464998	88	88
glpK	EGDe (NCBI)	complement(1573354..1574847)	39,35245177	1494	39,3524518	87	87
lmo1848	EGDe (NCBI)	complement(1924754..1925596)	68,94043459	843	68,9404346	86	86
groEL	EGDe (NCBI)	complement(2147684..2149312)	33,60215066	1629	33,6021507	81	81
eno	EGDe (NCBI)	complement(2526222..2527514)	38,67553545	1293	38,6755354	74	74
lmo2586	EGDe (NCBI)	complement(2665666..2668653)	15,15295557	2988	15,1529556	67	67
lmo2205	EGDe (NCBI)	complement(2293775..2294464)	60,72195406	690	60,7219541	62	62
ftsH	EGDe (NCBI)	226854..228929	19,2055966	2076	19,2055966	59	59
pgm	EGDe (NCBI)	complement(2527650..2529182)	26,00836174	1533	26,0083617	59	59
rpoC	EGDe (NCBI)	276728..280333	10,869396	3606	10,869396	58	58
lmo2196	EGDe (NCBI)	complement(2284539..2286215)	23,37211805	1677	23,372118	58	58
lmo2637	EGDe (NCBI)	complement(2709522..2710421)	43,55004662	900	43,5500466	58	58
clpX	EGDe (NCBI)	1291710..1292969	30,57084554	1260	30,5708455	57	57
sod	EGDe (NCBI)	complement(1472980..1473588)	63,25002525	609	63,2500252	57	57
cspB	EGDe (NCBI)	complement(2094877..2095077)	191,6381362	201	191,638136	57	57
dnaK	EGDe (NCBI)	complement(1505312..1507153)	20,54478219	1842	20,5447822	56	56
lmo2638	EGDe (NCBI)	2710869..2712755	19,69672083	1887	19,6967208	55	55
lmo2695	EGDe (NCBI)	2771623..2772612	37,54314364	990	37,5431436	55	55
clpC	EGDe (NCBI)	250592..253054	14,81605181	2463	14,8160518	54	54
lmo0641	EGDe (NCBI)	681543..683423	19,40028475	1881	19,4002848	54	54
lmo1468	EGDe (NCBI)	complement(1501415..1501861)	80,12563542	447	80,1256354	53	53
lmo1674	EGDe (NCBI)	complement(1727824..1728651)	43,25623072	828	43,2562307	53	53
tig	EGDe (NCBI)	1290241..1291524	27,3678991	1284	27,3678991	52	52
lmo1502	EGDe (NCBI)	complement(1531716..1532132)	84,26950228	417	84,2695023	52	52
rpsB	EGDe (NCBI)	complement(1707168..1707917)	46,85384327	750	46,8538433	52	52
acpP	EGDe (NCBI)	complement(1881041..1881274)	147,2846404	234	147,28464	51	51
clpB	EGDe (NCBI)	complement(2294555..2297155)	12,99070714	2601	12,9907071	50	50
lmo0134	EGDe (NCBI)	136714..136992	118,6847767	279	118,684777	49	49
cydB	EGDe (NCBI)	complement(2791967..2792980)	32,65587051	1014	32,6558705	49	49
ssb	EGDe (NCBI)	49934..50470	60,404611	537	60,404611	48	48
rplL	EGDe (NCBI)	267010..267372	89,35888735	363	89,3588873	48	48
inlA	EGDe (NCBI)	454534..456936	13,49865839	2403	13,4986584	48	48
PdhD	EGDe (NCBI)	1082822..1084225	22,62215066	1404	22,6221507	47	47
pflB	EGDe (NCBI)	1435635..1437866	14,23006251	2232	14,2300625	47	47
lmo1580	EGDe (NCBI)	1622583..1623047	66,85101707	465	66,8510171	46	46
lmo1255	EGDe (NCBI)	complement(1279721..1281205)	20,02300994	1485	20,0230099	44	44
cspL	EGDe (NCBI)	1387014..1387214	147,9311929	201	147,931193	44	44
pykA	EGDe (NCBI)	complement(1608166..1609923)	16,91363468	1758	16,9136347	44	44
tcsA	EGDe (NCBI)	1413646..1414719	27,05623201	1074	27,056232	43	43
dnaJ	EGDe (NCBI)	complement(1504037..1505170)	25,62468534	1134	25,6246853	43	43
rplJ	EGDe (NCBI)	266431..266931	55,30307387	501	55,3030739	41	41
lmo1541	EGDe (NCBI)	complement(1576411..1576728)	87,1284277	318	87,1284277	41	41
lmo1539	EGDe (NCBI)	complement(1574922..1575740)	33,00496144	819	33,0049614	40	40
tsf	EGDe (NCBI)	complement(1706204..1707088)	30,54357449	885	30,5435745	40	40
rpsD	EGDe (NCBI)	1639120..1639722	43,70694334	603	43,7069433	39	39

mecA	EGDe (NCBI)	complement(2278639..2279292)	40,29860373	654	40,2986037	39	39
kat	EGDe (NCBI)	complement(2871318..2872784)	17,9654307	1467	17,9654307	39	39
lmo0196	EGDe (NCBI)	200094..200402	83,10521117	309	83,1052112	38	38
lmo0415	EGDe (NCBI)	437482..438882	18,32941488	1401	18,3294149	38	38
rplA	EGDe (NCBI)	265494..266183	36,23729517	690	36,2372952	37	37
lmo2411	EGDe (NCBI)	complement(2481732..2483126)	17,92382342	1395	17,9238234	37	37
gyrA	EGDe (NCBI)	8065..10593	9,619595524	2529	9,61959552	36	36
lmo0788	EGDe (NCBI)	813451..817878	5,494118582	4428	5,49411858	36	36
lmo1883	EGDe (NCBI)	complement(1955598..1956656)	22,33444806	1059	22,3344481	35	35
sigB	EGDe (NCBI)	930671..931450	29,45692809	780	29,4569281	34	34
infC	EGDe (NCBI)	complement(1859272..1859787)	44,52791455	516	44,5279146	34	34
fur	EGDe (NCBI)	complement(2030744..2031196)	50,72053843	453	50,7205384	34	34
lmo2101	EGDe (NCBI)	2181329..2182216	25,87432873	888	25,8743287	34	34
lmo2449	EGDe (NCBI)	complement(2520415..2522796)	9,362144132	2382	9,36214413	33	33
lmo0098	EGDe (NCBI)	105951..106862	23,71145914	912	23,7114591	32	32
lmo0781	EGDe (NCBI)	complement(804887..805765)	23,83284886	879	23,8328489	31	31
rpsA	EGDe (NCBI)	complement(2012582..2013727)	18,28016942	1146	18,2801694	31	31
trxB	EGDe (NCBI)	complement(2553414..2554373)	21,82195224	960	21,8219522	31	31
lmo2696	EGDe (NCBI)	2772634..2773230	35,09057647	597	35,0905765	31	31
lmo0265	EGDe (NCBI)	287853..288992	17,78359436	1140	17,7835944	30	30
lmo2434	EGDe (NCBI)	complement(2502401..2503804)	14,43967063	1404	14,4396706	30	30
atpC	EGDe (NCBI)	complement(2605697..2606101)	50,05752486	405	50,0575249	30	30
lmo2673	EGDe (NCBI)	2745907..2746377	43,04309462	471	43,0430946	30	30
murC	EGDe (NCBI)	complement(1648511..1649854)	14,58148883	1344	14,5814888	29	29
sepA	EGDe (NCBI)	complement(2240068..2241969)	10,30363879	1902	10,3036388	29	29
rplK	EGDe (NCBI)	265029..265454	44,41724037	426	44,4172404	28	28
lmo0401	EGDe (NCBI)	421469..424096	7,200054945	2628	7,20005495	28	28
clpE	EGDe (NCBI)	complement(1026871..1029045)	8,699652596	2175	8,6996526	28	28
lmo1578	EGDe (NCBI)	1620091..1621188	17,23291839	1098	17,2329184	28	28
lmo1579	EGDe (NCBI)	complement(1621230..1622342)	17,00066882	1113	17,0006688	28	28
lmo1694	EGDe (NCBI)	1757771..1758673	20,95431273	903	20,9543127	28	28
lmo2085	EGDe (NCBI)	complement(2162323..2164011)	11,20292741	1689	11,2029274	28	28
atpD	EGDe (NCBI)	complement(2606123..2607544)	13,30643066	1422	13,3064307	28	28
recA	EGDe (NCBI)	1425419..1426465	17,42690335	1047	17,4269034	27	27
uvrA	EGDe (NCBI)	complement(2562591..2565461)	6,355265695	2871	6,35526569	27	27
pbpB	EGDe (NCBI)	complement(2121483..2123738)	7,78820533	2256	7,78820533	26	26
ptsH	EGDe (NCBI)	1032924..1033190	63,27496119	267	63,2749612	25	25
pycA	EGDe (NCBI)	1099266..1102706	4,909739796	3441	4,9097398	25	25
lmo1340	EGDe (NCBI)	1367559..1368650	15,47107568	1092	15,4710757	25	25
rpmA	EGDe (NCBI)	complement(1576107..1576397)	58,05640769	291	58,0564077	25	25
lmo1068	EGDe (NCBI)	1095975..1096835	18,83697799	861	18,836978	24	24
pflC	EGDe (NCBI)	1437943..1438689	21,71169753	747	21,7116975	24	24
purB	EGDe (NCBI)	complement(1845044..1846336)	12,5434169	1293	12,5434169	24	24
secY	EGDe (NCBI)	complement(2695476..2696771)	12,51438121	1296	12,5143812	24	24
qoxB	EGDe (NCBI)	17344..19323	7,849930034	1980	7,84993003	23	23
nifJ	EGDe (NCBI)	855759..859406	4,260652815	3648	4,26065281	23	23
rpsU	EGDe (NCBI)	complement(1501881..1502054)	89,32679004	174	89,32679	23	23
spxA	EGDe (NCBI)	complement(2279524..2279919)	39,24965017	396	39,2496502	23	23
lmo2828	EGDe (NCBI)	complement(2915744..2916097)	43,90638833	354	43,9063883	23	23
rpsR	EGDe (NCBI)	50514..50753	61,94618701	240	61,946187	22	22
lmo1376	EGDe (NCBI)	1400941..1402359	10,47715637	1419	10,4771564	22	22
secA	EGDe (NCBI)	complement(2587300..2589813)	5,913717137	2514	5,91371714	22	22
lmo2668	EGDe (NCBI)	complement(2740888..2742957)	7,18216661	2070	7,18216661	22	22
lmo0010	EGDe (NCBI)	12918..13886	14,645313	969	14,645313	21	21
lmo0392	EGDe (NCBI)	415224..416168	15,01725746	945	15,0172575	21	21
lmo0653	EGDe (NCBI)	complement(695496..696416)	15,40858664	921	15,4085866	21	21
lmo1350	EGDe (NCBI)	1375462..1376928	9,673693454	1467	9,67369345	21	21
ccpA	EGDe (NCBI)	complement(1642865..1643872)	14,07867887	1008	14,0786789	21	21
trmD	EGDe (NCBI)	complement(1864657..1865394)	19,22941504	738	19,229415	21	21
lmo2384	EGDe (NCBI)	2460090..2460683	23,89109141	594	23,8910914	21	21
gltX	EGDe (NCBI)	256983..258458	9,156864303	1476	9,1568643	20	20
hrcA	EGDe (NCBI)	complement(1507804..1508841)	13,02074346	1038	13,0207435	20	20
lmo1830	EGDe (NCBI)	1905952..1906551	22,52588619	600	22,5258862	20	20
lmo1992	EGDe (NCBI)	2065698..2066417	18,77157182	720	18,7715718	20	20
lmo2102	EGDe (NCBI)	2182218..2182784	23,8369166	567	23,8369166	20	20
lmo2572	EGDe (NCBI)	complement(2650231..2650755)	25,74386993	525	25,7438699	20	20
lmo2672	EGDe (NCBI)	complement(2744985..2745791)	16,74787077	807	16,7478708	20	20
lmo0133	EGDe (NCBI)	136469..136702	54,8707484	234	54,8707484	19	19

lmo0515	EGDe (NCBI)	549438..549869	29,72165538	432	29,7216554	19	19
lmo0994	EGDe (NCBI)	complement(1024761..1025129)	34,79608435	369	34,7960844	19	19
lmo1718	EGDe (NCBI)	complement(1779714..1780802)	11,79040875	1089	11,7904087	19	19
adk	EGDe (NCBI)	complement(2694769..2695416)	19,81443692	648	19,8144369	19	19
lmo2713	EGDe (NCBI)	complement(2786435..2787373)	13,67386062	939	13,6738606	19	19
lmo0050	EGDe (NCBI)	52630..53925	9,385785911	1296	9,38578591	18	18
lmo0727	EGDe (NCBI)	756738..758543	6,735314806	1806	6,73531481	18	18
lmo0796	EGDe (NCBI)	complement(823638..824168)	22,90768087	531	22,9076809	18	18
lmo0953	EGDe (NCBI)	987274..987501	53,35078307	228	53,3507831	18	18
lmo1301	EGDe (NCBI)	complement(1329696..1330217)	23,30264088	522	23,3026409	18	18
rpsT	EGDe (NCBI)	1513917..1514171	47,70187663	255	47,7018766	18	18
aspS	EGDe (NCBI)	complement(1550854..1552629)	6,849087016	1776	6,84908702	18	18
lmo1780	EGDe (NCBI)	complement(1855952..1857184)	9,865351614	1233	9,86535161	18	18
rplS	EGDe (NCBI)	complement(1861907..1862251)	35,25790881	345	35,2579088	18	18
pgi	EGDe (NCBI)	complement(2444231..2445583)	8,990375861	1353	8,99037586	18	18
pgk	EGDe (NCBI)	complement(2529985..2531175)	10,21324814	1191	10,2132481	18	18
rpsE	EGDe (NCBI)	complement(2697463..2697966)	24,13487806	504	24,1348781	18	18
guaB	EGDe (NCBI)	complement(2837944..2839410)	8,291737246	1467	8,29173725	18	18
rpoB	EGDe (NCBI)	273003..276557	3,231561731	3555	3,23156173	17	17
lmo0722	EGDe (NCBI)	751598..753328	6,636742897	1731	6,6367429	17	17
pdhC	EGDe (NCBI)	1081183..1082817	7,026423214	1635	7,02642321	17	17
lmo1059	EGDe (NCBI)	1086100..1086630	21,63503193	531	21,6350319	17	17
infB	EGDe (NCBI)	1353696..1356035	4,909488015	2340	4,90948801	17	17
lmo1509	EGDe (NCBI)	complement(1539979..1542375)	4,792741742	2397	4,79274174	17	17
lmo1687	EGDe (NCBI)	complement(1752413..1752940)	21,75795825	528	21,7579582	17	17
asnC	EGDe (NCBI)	complement(1969037..1970329)	8,884920305	1293	8,88492031	17	17
lmo2173	EGDe (NCBI)	complement(2255034..2256401)	8,397808446	1368	8,39780845	17	17
lmo2560	EGDe (NCBI)	complement(2639930..2640466)	21,39329973	537	21,3932997	17	17
rplC	EGDe (NCBI)	complement(2705082..2705711)	18,2352412	630	18,2352412	17	17
lmo2738	EGDe (NCBI)	complement(2813949..2815283)	8,605394722	1335	8,60539472	17	17
rsbX	EGDe (NCBI)	931451..932050	18,02070895	600	18,0207089	16	16
lmo0937	EGDe (NCBI)	complement(973509..973670)	66,74336647	162	66,7433665	16	16
lmo1027	EGDe (NCBI)	complement(1055393..1057060)	6,482269406	1668	6,48226941	16	16
guaA	EGDe (NCBI)	1129945..1131501	6,944396512	1557	6,94439651	16	16
lmo1434	EGDe (NCBI)	complement(1465638..1467305)	6,482269406	1668	6,48226941	16	16
alaS	EGDe (NCBI)	complement(1532493..1535132)	4,09561567	2640	4,09561567	16	16
pbpA	EGDe (NCBI)	1964489..1966972	4,352828248	2484	4,35282825	16	16
lmo1930	EGDe (NCBI)	complement(2004977..2005942)	11,19298692	966	11,1929869	16	16
ftsZ	EGDe (NCBI)	complement(2112492..2113667)	9,194239259	1176	9,19423926	16	16
mscL	EGDe (NCBI)	2145127..2145513	27,93908364	387	27,9390836	16	16
lmo2386	EGDe (NCBI)	2461219..2461692	22,81102399	474	22,811024	16	16
lmo2425	EGDe (NCBI)	complement(2494891..2495268)	28,60429992	378	28,6042999	16	16
ami	EGDe (NCBI)	2635167..2637920	3,926080381	2754	3,92608038	16	16
lmo0027	EGDe (NCBI)	29100..31004	5,321075477	1905	5,32107548	15	15
inlH	EGDe (NCBI)	284365..286011	6,154613712	1647	6,15461371	15	15
lmo0292	EGDe (NCBI)	316873..318375	6,744277301	1503	6,7442773	15	15
lmo0540	EGDe (NCBI)	578886..580079	8,489655597	1194	8,4896556	15	15
lmo0553	EGDe (NCBI)	complement(591412..592047)	15,93812702	636	15,938127	15	15
lmo0739	EGDe (NCBI)	769639..771012	7,377473641	1374	7,37747364	15	15
lmo0956	EGDe (NCBI)	989099..990232	8,938843724	1134	8,93884372	15	15
trxA	EGDe (NCBI)	1259530..1259841	32,48925892	312	32,4892589	15	15
lmo1529	EGDe (NCBI)	complement(1564776..1565105)	30,71711753	330	30,7171175	15	15
lmo1576	EGDe (NCBI)	complement(1617901..1619214)	7,714344584	1314	7,71434458	15	15
menE	EGDe (NCBI)	complement(1725584..1726993)	7,189112612	1410	7,18911261	15	15
rpsP	EGDe (NCBI)	complement(1867427..1867699)	37,13058162	273	37,1305816	15	15
lmo1849	EGDe (NCBI)	complement(1925600..1926322)	14,02026111	723	14,0202611	15	15
lmo1859	EGDe (NCBI)	complement(1934321..1934758)	23,14303375	438	23,1430338	15	15
hemH	EGDe (NCBI)	complement(2300334..2301263)	10,89962235	930	10,8996223	15	15
lmo2220	EGDe (NCBI)	complement(2307755..2308696)	10,76077366	942	10,7607737	15	15
lmo2389	EGDe (NCBI)	complement(2463969..2465180)	8,363571603	1212	8,3635716	15	15
lmo2585	EGDe (NCBI)	complement(2665190..2665666)	21,25083602	477	21,250836	15	15
lmo0650	EGDe (NCBI)	691581..694271	3,515745893	2691	3,51574589	14	14
lmo0721	EGDe (NCBI)	complement(750680..751327)	14,60011142	648	14,6001114	14	14
lmo0738	EGDe (NCBI)	767766..769619	5,102951563	1854	5,10295156	14	14
lmo0782	EGDe (NCBI)	complement(805784..806596)	11,63698917	813	11,6369892	14	14
PdhB	EGDe (NCBI)	1080095..1081072	9,673693454	978	9,67369345	14	14
glnA	EGDe (NCBI)	1326879..1328213	7,086795654	1335	7,08679565	14	14
tkt	EGDe (NCBI)	1331813..1333807	4,742291828	1995	4,74229183	14	14
lmo1501	EGDe (NCBI)	complement(1531398..1531700)	31,22400065	303	31,2240007	14	14
pfkA	EGDe (NCBI)	complement(1610206..1611165)	9,855075206	960	9,85507521	14	14

lmo1620	EGDe (NCBI)	complement(1662570..1663982)	6,695592497	1413	6,6955925	14	14
pflA	EGDe (NCBI)	1991047..1993326	4,14950535	2280	4,14950535	14	14
lmo2483	EGDe (NCBI)	complement(2558130..2559068)	10,07547625	939	10,0754762	14	14
lmo2571	EGDe (NCBI)	complement(2649598..2650230)	14,94608562	633	14,9460856	14	14
rpsG	EGDe (NCBI)	complement(2729457..2729927)	20,08677749	471	20,0867775	14	14
lmo2697	EGDe (NCBI)	2773234..2773608	25,22899253	375	25,2289925	14	14
cydD	EGDe (NCBI)	complement(2788504..2790243)	5,437282872	1740	5,43728287	14	14
dnaC	EGDe (NCBI)	57286..58638	6,493049233	1353	6,49304923	13	13
lmo0400	EGDe (NCBI)	420335..421447	7,893167666	1113	7,89316767	13	13
lmo0402	EGDe (NCBI)	424130..426064	4,540101092	1935	4,54010109	13	13
lmo0555	EGDe (NCBI)	593528..595006	5,939888852	1479	5,93988885	13	13
rsbW	EGDe (NCBI)	930220..930693	18,53395699	474	18,533957	13	13
lmo0964	EGDe (NCBI)	complement(996739..997557)	10,72661247	819	10,7266125	13	13
recN	EGDe (NCBI)	1390694..1392385	5,192136887	1692	5,19213689	13	13
lmo1395	EGDe (NCBI)	1422236..1423165	9,446339368	930	9,44633937	13	13
lmo1527	EGDe (NCBI)	complement(1561978..1564242)	3,878629409	2265	3,87862941	13	13
rplU	EGDe (NCBI)	complement(1576747..1577055)	28,43073014	309	28,4307301	13	13
lmo1602	EGDe (NCBI)	complement(1645958..1646413)	19,26556055	456	19,2655606	13	13
lmo1606	EGDe (NCBI)	complement(1650149..1652500)	3,735159699	2352	3,7351597	13	13
lmo1819	EGDe (NCBI)	complement(1892603..1893478)	10,02864796	876	10,028648	13	13
lmo2471	EGDe (NCBI)	complement(2545487..2546503)	8,63824544	1017	8,63824544	13	13
atpI	EGDe (NCBI)	complement(2612205..2612603)	22,01778349	399	22,0177835	13	13
lmo2573	EGDe (NCBI)	complement(2650758..2651756)	8,793889502	999	8,7938895	13	13
rpoA	EGDe (NCBI)	complement(2692097..2693041)	9,296397473	945	9,29639747	13	13
rpsL	EGDe (NCBI)	complement(2729958..2730371)	21,22003771	414	21,2200377	13	13
cydC	EGDe (NCBI)	complement(2790243..2791967)	5,092809051	1725	5,09280905	13	13
qoxC	EGDe (NCBI)	19311..19922	13,25052129	612	13,2505213	12	12
lmo0043	EGDe (NCBI)	48074..49306	6,576901076	1233	6,57690108	12	12
metS	EGDe (NCBI)	175766..177760	4,064821567	1995	4,06482157	12	12
lmo0242	EGDe (NCBI)	262064..262576	15,80763943	513	15,8076394	12	12
lmo0584	EGDe (NCBI)	623251..624423	6,913315453	1173	6,91331545	12	12
lmo0625	EGDe (NCBI)	complement(661470..662171)	11,55173651	702	11,5517365	12	12
lmo0866	EGDe (NCBI)	905962..907524	5,18830392	1563	5,18830392	12	12
lmo0931	EGDe (NCBI)	967784..968779	8,141886573	996	8,14188657	12	12
lmo0958	EGDe (NCBI)	990968..991690	11,21620889	723	11,2162089	12	12
rpsO	EGDe (NCBI)	1358834..1359103	30,03451491	270	30,0345149	12	12
valS	EGDe (NCBI)	complement(1586201..1588852)	3,057812604	2652	3,0578126	12	12
lmo1575	EGDe (NCBI)	complement(1616945..1617880)	8,663802379	936	8,66380238	12	12
gatB	EGDe (NCBI)	complement(1823494..1824924)	5,666889606	1431	5,66688961	12	12
groES	EGDe (NCBI)	complement(2149348..2149632)	28,45375097	285	28,453751	12	12
lmo2256	EGDe (NCBI)	complement(2345567..2346088)	15,53509392	522	15,5350939	12	12
lmo2475	EGDe (NCBI)	complement(2549462..2551192)	4,684759692	1731	4,68475969	12	12
rplD	EGDe (NCBI)	complement(2704433..2705056)	12,99570357	624	12,9957036	12	12
lmo2674	EGDe (NCBI)	2746423..2746878	17,78359436	456	17,7835944	12	12
dnaX	EGDe (NCBI)	complement(2777844..2779583)	4,660528176	1740	4,66052818	12	12
lmo2720	EGDe (NCBI)	2795420..2796997	5,138985442	1578	5,13898544	12	12
lmo0305	EGDe (NCBI)	329923..330999	6,902082118	1077	6,90208212	11	11
lmo0471	EGDe (NCBI)	507723..508733	7,352663147	1011	7,35266315	11	11
lmo0763	EGDe (NCBI)	788642..789514	8,514939795	873	8,5149398	11	11
lmo0794	EGDe (NCBI)	complement(821886..822527)	11,57872654	642	11,5787265	11	11
lmo0957	EGDe (NCBI)	990248..990952	10,54403183	705	10,5440318	11	11
lmo1084	EGDe (NCBI)	1118082..1118912	8,945297763	831	8,94529776	11	11
lmo1232	EGDe (NCBI)	1257092..1259449	3,15247771	2358	3,15247771	11	11
lmo1349	EGDe (NCBI)	1374119..1375465	5,51859127	1347	5,51859127	11	11
lmo1394	EGDe (NCBI)	1421454..1422185	10,15511262	732	10,1551126	11	11
lmo1424	EGDe (NCBI)	complement(1455316..1456662)	5,51859127	1347	5,51859127	11	11
menD	EGDe (NCBI)	complement(1728653..1730395)	4,264797729	1743	4,26479773	11	11
lmo1690	EGDe (NCBI)	1755143..1756123	7,577515231	981	7,57751523	11	11
lmo2067	EGDe (NCBI)	complement(2146449..2147426)	7,600759142	978	7,60075914	11	11
lmo2113	EGDe (NCBI)	2192693..2193448	9,832728097	756	9,8327281	11	11
fabG_2	EGDe (NCBI)	complement(2257754..2258515)	9,755305041	762	9,75530504	11	11
lmo2267	EGDe (NCBI)	complement(2352616..2356323)	2,004730971	3708	2,00473097	11	11
lmo2391	EGDe (NCBI)	2466713..2467342	11,79927372	630	11,7992737	11	11
lmo2413	EGDe (NCBI)	complement(2483589..2484815)	6,058306798	1227	6,0583068	11	11
lmo2462	EGDe (NCBI)	complement(2535342..2536268)	8,018923885	927	8,01892388	11	11
lmo2515	EGDe (NCBI)	complement(2593837..2594523)	10,82029467	687	10,8202947	11	11
atpH	EGDe (NCBI)	complement(2610056..2610595)	13,76581934	540	13,7658193	11	11
lmo2671	EGDe (NCBI)	complement(2744571..2744939)	20,14510147	369	20,1451015	11	11
lmo2760	EGDe (NCBI)	complement(2840286..2841896)	4,614241118	1611	4,61424112	11	11
rpmH	EGDe (NCBI)	complement(2943569..2943703)	55,06327734	135	55,0632773	11	11

lmo0052	EGDe (NCBI)	54839..56812	3,423386958	1974	3,42338696	10	10
lmo0135	EGDe (NCBI)	137323..138897	4,290644988	1575	4,29064499	10	10
lmo0192	EGDe (NCBI)	195793..196611	8,251240361	819	8,25124036	10	10
lysS	EGDe (NCBI)	235524..237020	4,514205648	1497	4,51420565	10	10
lmo0231	EGDe (NCBI)	249541..250563	6,605831726	1023	6,60583173	10	10
lmo0291	EGDe (NCBI)	315945..316775	8,132088876	831	8,13208888	10	10
lmo0592	EGDe (NCBI)	633709..634251	12,44524099	543	12,445241	10	10
lmo0626	EGDe (NCBI)	complement(662257..663936)	4,022479676	1680	4,02247968	10	10
lmo1087	EGDe (NCBI)	1121805..1122830	6,586516428	1026	6,58651643	10	10
lmo1218	EGDe (NCBI)	1239039..1239797	8,903512326	759	8,90351233	10	10
pheT_1	EGDe (NCBI)	1242100..1244508	2,805216212	2409	2,80521621	10	10
lmo1261	EGDe (NCBI)	complement(1285610..1286749)	5,927864786	1140	5,92786479	10	10
lmo1303	EGDe (NCBI)	1331018..1331347	20,47807835	330	20,4780784	10	10
pnpA	EGDe (NCBI)	1359352..1361523	3,111310247	2172	3,11131025	10	10
lmo1386	EGDe (NCBI)	1410148..1412421	2,971752795	2274	2,97175279	10	10
lmo1423	EGDe (NCBI)	complement(1454347..1455177)	8,132088876	831	8,13208888	10	10
era	EGDe (NCBI)	complement(1495899..1496804)	7,45890271	906	7,45890271	10	10
citC	EGDe (NCBI)	complement(1604613..1605875)	5,35056679	1263	5,35056679	10	10
ackA	EGDe (NCBI)	complement(1623099..1624292)	5,659770398	1194	5,6597704	10	10
lmo1678	EGDe (NCBI)	complement(1732926..1734779)	3,644965402	1854	3,6449654	10	10
glmM	EGDe (NCBI)	complement(2197763..2199115)	4,994653256	1353	4,99465326	10	10
lmo2199	EGDe (NCBI)	complement(2288571..2288987)	16,20567351	417	16,2056735	10	10
lmo2202	EGDe (NCBI)	complement(2290989..2291927)	7,196768749	939	7,19676875	10	10
lmo2230	EGDe (NCBI)	2320416..2320841	15,86330013	426	15,8633001	10	10
lmo2341	EGDe (NCBI)	complement(2409875..2410993)	6,039111578	1119	6,03911158	10	10
lmo2373	EGDe (NCBI)	complement(2449878..2450192)	21,45322494	315	21,4532249	10	10
lmo2417	EGDe (NCBI)	complement(2488379..2489209)	8,132088876	831	8,13208888	10	10
rplM	EGDe (NCBI)	complement(2684665..2685102)	15,42868917	438	15,4286892	10	10
rplO	EGDe (NCBI)	complement(2696771..2697211)	15,3237321	441	15,3237321	10	10
rplB	EGDe (NCBI)	complement(2703275..2704108)	8,102836757	834	8,10283676	10	10
serS	EGDe (NCBI)	complement(2821912..2823195)	5,26305752	1284	5,26305752	10	10
* Reads number ≥ 10							
